# A Critical Review of Bovine Viral Diarrhea Virus: Spotlights on Host Plasticity and Potential Spillover Events

**DOI:** 10.3390/v17091221

**Published:** 2025-09-07

**Authors:** Eaftekhar Ahmed Rana, M. Asaduzzaman Prodhan, Joshua W. Aleri, Syeda Hasina Akter, Henry Annandale, Sam Abraham, Subir Sarker, Jully Gogoi-Tiwari, Jasim Muhammad Uddin

**Affiliations:** 1 School of Veterinary Medicine, Murdoch University, Perth, WA 6150, Australia; eaftekhar.rana@murdoch.edu.au (E.A.R.); asad.prodhan@murdoch.edu.au (M.A.P.); hasina.akter@murdoch.edu.au (S.H.A.); henry.annandale@murdoch.edu.au (H.A.); jully.gogoitiwari@murdoch.edu.au (J.G.-T.); 2 Department of Microbiology and Veterinary Public Health, Chattogram Veterinary and Animal Sciences University, Zakir Hossain Road, Khulsi, Chattogram 4225, Bangladesh; 3 School of Veterinary Science, The University of Queensland, Gatton, QLD 4343, Australia; 4 Centre for Biosecurity and One Health, Harry Butler Institute, Murdoch University, Perth, WA 6150, Australia; s.abraham@murdoch.edu.au; 5 Biomedical Sciences & Molecular Biology, College of Medicine and Dentistry, James Cook University, Townsville, QLD 48114, Australia; subir.sarker@jcu.edu.au

**Keywords:** BVDV, cross-species, spillover, host tropism, plasticity, transmission cycle

## Abstract

The bovine viral diarrhea virus (BVDV) infects a wide range of domestic and wild mammals. This review hypothesized that there might be cross-species transmission of BVDV. Therefore, the aim was to explore the BVDV-5′ UTR and N-pro sequence-based evidence to understand host plasticity among different animals. A total of 146 unique BVDV sequences retrieved from GenBank, originating from 12 distinct mammalian species that are submitted from 55 countries, were analyzed. The phylogenetic analysis revealed that all three BVDV species exhibited genetic relatedness infecting diverse animal species. BVDV-1 sequences obtained from cattle, buffalo, and pigs and BVDV-2 and HoBi-like pestivirus sequences from cattle, goats, and sheep showed a genetic resemblance. Surprisingly, cattle and buffalo in China, cattle and yak in Mongolia, cattle and wild boar in Serbia, cattle and deer in Mexico, cattle and alpacas in Canada, goats and pigs in the USA, and sheep and buffalo in Argentina were infected with BVDV-1 within the same county and strongly positioned in the same cluster, indicating potential spillover with host tropism. Moreover, BVDV sequences isolated from various neighboring countries clustered closely, suggesting potential cross-border transmission events. Based on genomic evidence, the BVDV transmission cycle could be depicted, where cattle act as a primary source of infection, while other domestic and wild animals maintain the infection ecology within their habitat due to virus tropism.

## 1. Introduction

Bovine viral diarrhea virus (BVDV) is an enveloped positive-sense, single-stranded RNA virus with an approximately 12.3 kb genome and a single open reading frame (ORF). It belongs to the genus *Pestivirus* (family *Flaviviridae*), affecting multiple organ systems in susceptible animals, and the disease is characterized by long-term immunosuppression, poor growth and production, different reproductive disorders, gastrointestinal and respiratory diseases, and non-visible teratogenic damage [1,2]. BVDV is considered a Class III infectious disease for livestock and causes a high economic burden in cattle herds as well as other animal production systems [3]. To date, based on genomic analysis of the 5′-untranslated region (UTR), envelope glycoprotein (E2), and N-terminal autoprotease (N-pro), BVDV is classified into three distinct species, namely BVDV-1 (*Pestivirus bovis*), BVDV-2 (*Pestivirus tauri*), and HoBi-like pestivirus (*Pestivirus brazilense*) (previous nomenclature was BVDV-3) [1,4,5]. In addition, genetically distinct species (previously genotypes) like pronghorn pestivirus (*Pestivirus antilocaprae*), giraffe pestivirus (*Pestivirus giraffae*), and reindeer strain V60 were also reported in wildlife environments [4,6]. BVDV is capable of inducing persistent infection (PI) in animals and maintaining viral circulation within herd environments through continuous viral shedding [7]. Based on the cytopathic effects on susceptible cell lines, BVDV exists in two biotypes, cytopathic and non-cytopathic [1,4]. The non-cytopathic biotype is typically associated with PI and plays a triggering role in the development of mucosal disease (MD) when co-infection with the cytopathic biotype occurs in the host [1,8]. However, the genus *Pestivirus*, especially BVDV, infects a wide range of domestic and wild animal species within the order Artiodactyla [1,9]. Serological and antigenic studies found that several mammalian species including cattle, buffalo, sheep, goats, pigs, deer, bison, yak, camel, alpacas, llamas, and giraffes are susceptible to BVDV infection [1,2,7,10]. These studies suggest the potential for host jumps and cross-species spillover of the infection.

Notably, virus spillover is usually an uncertain and accidental event in which a contagious viral pathogen transmits from a reservoir animal to a susceptible host population [11]. The consequence of virus spillover in a naive host may lead to an initial spark for the evolution of a new pathotype, variant, or genotype or remain the same, which could break the species’ barriers, aggravate the infection process, and/or facilitate transmission dynamics [11]. Even the new spillover viral pathogen becomes adapted in the novel host and plays a significant role in the viral survival and replication strategy [6], establishing new epidemics of disease between diverse species. Understanding the true reservoir distribution, ecology, epidemiology, and evolutionary history of potential spillover pathogens are essential for epidemic preparedness and management interventions to predict the outcome of spillover events.

Furthermore, the host tropism and the detection rate of BVDV spillover to novel domestic and wildlife hosts have been increasing in recent years [1,6]. Unfortunately, the BVDV infection-to-disease progression in diverse host bodies is not well characterized, but it continuously poses a threat to global animal health security. It is also of utmost importance to understand host–BVDV interactions, including host adaptation, tissue tropism, and cross-species transmission across a wide range of mammalian species. Such information is essential for developing species-specific control strategies, including targeted vaccination and management practices, especially in mixed-species farming systems where BVDV spillover is likely to occur. BVDV isolates from diverse animal species were sequenced by several researchers from different countries, but no study has analyzed them together to explore the host-jump and transmission dynamics at the interspecies interface. Therefore, this review aims to utilize 5’-UTR and N-pro gene sequences from GenBank to assess the genomic relationships of BVDV through phylogenetic analysis. Additionally, it represents the current understanding of cross-species spillover events among diverse domestic and wild mammalian hosts and highlights directions for future research.

## 2. Materials and Methods

### 2.1. Preparation of Genomic Dataset

To analyze the genetic relatedness among different BVDV species (previously known as genotypes), we primarily selected and retrieved 5′ UTR sequences that were submitted from different countries of the world in different time intervals from diverse animal species, including both domestic and wildlife hosts. Moreover, we included the N-pro gene only for those countries or species where 5′ UTR sequences were not available in the GenBank database. We identified the BVDV genome sequence that represents the highest blast score and query coverage using its GenBank ID, species or genotype, host name, and country of origin in the National Center for Biotechnology Information (NCBI) database (https://blast.ncbi.nlm.nih.gov/Blast.cgi, accessed repeatedly between 4 July 2024 and 30 June 2025).

### 2.2. Sequence Selection Criteria and Rationale

We selected a single sequence per BVDV species from each country for each domestic and wild mammalian species that was submitted and publicly available in the NCBI GenBank database (Table 1). Where the virus sequence from the natural infection of an animal species is not available, the sequence data from experimental BVDV infections in distinct animal hosts was included to understand the virus’s host tropism and susceptibility. The selection strategy aimed to prevent redundancy and ensure a diverse representation of the BVDV species, avoiding multiple sequences from the same host and country. This approach reduces potential biases in phylogenetic clustering that could result from self-alignments. We hypothesized that phylogenetic analysis of every single sequence asserted tenable information to understand the evolutionary dynamics as well as cross-species spillover evidence of BVDV. This review did not consider any BVDV seropositive animal cases or any cases that were confirmed solely by molecular testing without sequencing. Although several countries reported the existence of BVDV in various host species without sequencing data, we did not incorporate these as evidence of cross-species spillover.

### 2.3. Description of Different BVDV Species Sequence Data

**Figure 1:**
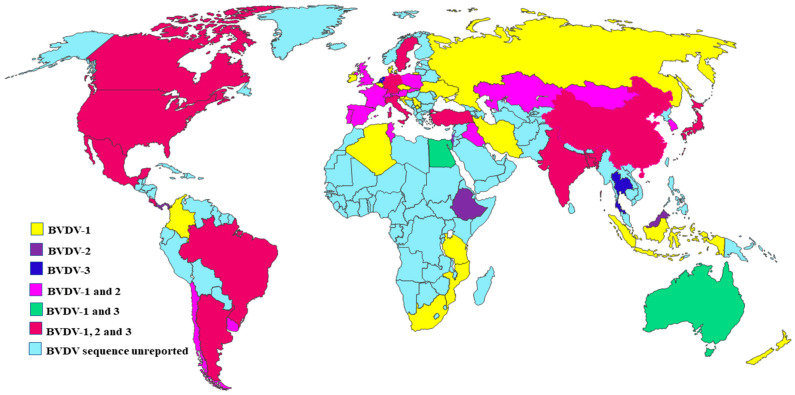
The global map represents the distribution and diversity of BVDV species based on 5′-UTR and N-pro gene sequences retrieved from the NCBI database, submitted by different countries around the world.

A total of 146 three BVDV species sequences [83 BVDV-1, 41 BVDV-2, and 22 HoBi like pestivirus] from 11 different animal species, submitted from 55 countries (14 Asian, 22 European, 4 North American, 6 South American, 7 African, and 2 Oceanian countries) (Figure 1) were retrieved from the NCBI GenBank database (Table 1). In addition, two BVDV sequences reported from Kosovo and Israel were obtained from published research articles as they were not submitted to the GenBank database (Table 1). Among the sequence data, 134 were specific to the 5′ untranslated region (5′-UTR) and 12 were specific to the N-pro gene of BVDV. Seven domestic animal species were found to be positive for BVDV infection, such as cattle (94 sequences included from 53 different countries), buffalo (10 sequences from 7 countries), sheep (11 sequences from 8 countries), goats (10 sequences from 8 countries), pigs (7 sequences from 6 countries), alpacas (2 sequences from 2 countries), and camels (single sequence from China). In addition, 5 wild animals, such as wild boar (2 sequences from 2 countries), deer (6 sequences from 6 countries), yak (3 sequences from 2 countries), bison (single sequence from Germany), and a single sequence from a bat species (OR439364.1), were found to be positive for BVDV (Table 1). One giraffe pestivirus-2 isolate (OR425033.1) from cattle in Egypt was described in the current review but was not included in the phylogenetic analysis due to it being a distinct species of pestivirus.

### 2.4. Phylogenetic Analysis and Construction of Phylogenetic Tree

All the retrieved BVDV nucleotide sequences, particularly 5′ UTR (approximately 293 bp) and N-pro genes (approximately 504 bp) were aligned separately using the MUSCLE (Multiple Sequence Comparison by Log-Expectation) program [12]. Furthermore, a neighbor-joining algorithm was selected to perform phylogenetic analysis using the MEGA-11 software program [13,14]. Subsequently, the evolutionary distances of phylogeny were measured using the Kimura 2-parameter method with default parameters [15]. Finally, the robustness, as well as the significance, of each phylogeny branch order were calculated using the bootstrapping method by applying 1000 replicates. In addition, the genomic similarity percentages were determined using the distance matrix algorithm of the MEGA-11 software program described by Kumar et al. [16], and the bootstrap values of the phylogenetic tree indicated each node that arises from the distinct genome sequence of BVDV.

## 3. Results

### 3.1. Genetic Variability and Phylogenetic Evidence of BVDV-1 (P. bovis) Intra- and Cross-Species Spillover

**Figure 2:**
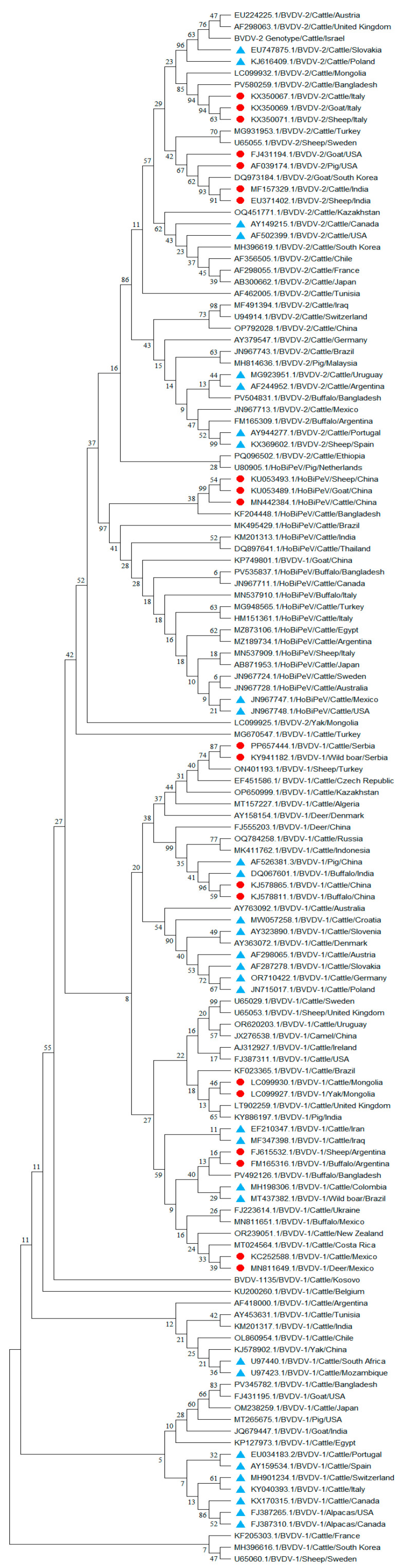
Phylogenetic tree illustrates the genetic relatedness of different species of BVDV based on 5′-UTR fragments isolated from different animal species across the globe. The phylogenetic tree was constructed using the neighbor-joining method performed in MEGA 11 software with bootstrap values as 1000 replicates, and distances were calculated using Kimura model. Branch lengths of the tree are proportional to genetic distances. The BVDV sequences incorporated in the current analysis are identified by the GenBank accession number (Table 1), except the sequence reported from Kosovo and Israel (sequence was not submitted in NCBI GenBank). Each bar of the tree indicates the GenBank accession number, BVDV species, host name, and country of origin, sequentially. The circular, solid red color represents the BVDV cross-species infection within the countries, and the triangular, solid cyan color indicates neighboring border countries positioned in the same cluster and clade based on BVDV genetic relatedness.

**Figure 3:**
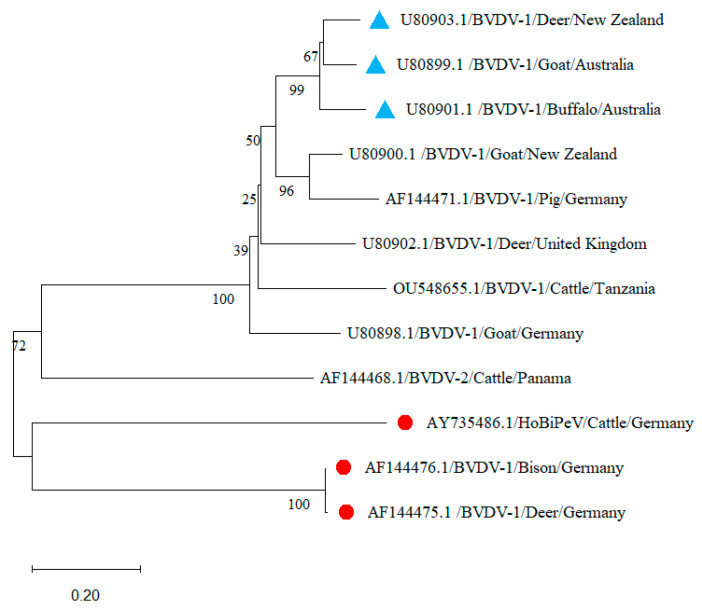
Phylogenetic tree depicts the genetic relatedness of different species of BVDV based on N-pro gene fragments isolated from different animal species across the globe. The BVDV sequences incorporated in the current analysis are identified by the GenBank accession number (Table 1). Each bar of the tree indicates the GenBank accession number, BVDV species, host name, and country of origin, sequentially. The circular, solid red color represents the BVDV cross-species infection within the countries, and the triangular, solid cyan color indicates neighboring border countries positioned in the same cluster and clade based on BVDV genetic relatedness.

BVDV-1 exhibits extensive genetic diversity across different host species worldwide (Figure 1), as supported by nucleotide sequence variability and distribution across countries (Figure 2 and Figure 3). The current phylogenetic cladogram shows that the majority of BVDV-1 derived from diverse animal species are clustered at the same clade, which indicates strong genetic relatedness and potential cross-species circulation, rather than separation into host-specific clusters (Figure 2). In addition, the BVDV-1 sequence detected from cattle in Russia (OQ784258.1)–Indonesia (MK411762.1), Germany (OR710422.1)–Poland (JN715017.1), Slovakia (AF287278.1)–Austria (AF298065.1), Slovenia (AY323890.1)–Denmark (AY363072.1), South Africa (U97440.1)–Mozambique (U97423.1), Iran (EF210347.1)–Iraq (MF347398.1), India (KM201317.1)–Tunisia (AY453631.1), and Portugal (EU034183.2)–Spain (AY159534.1), as well as Switzerland (MH901234.1)–Italy (KY040393.1) aligned within the same clade with a high range of genomic relatedness. Surprisingly, BVDV-1 was isolated from distinct domestic animal species, but they were positioned in the same clade due to their high degree of genetic similarity (Figure 2 and Figure 3), such as sheep–cattle [from the United Kingdom (U65053.1) and Sweden (U65029.1)]; camel–cattle [from China (JX276538.1) and Uruguay (OR620203.1)]; pig–cattle [from India (KY886197.1) and the UK(LT902259.1)]; cattle–buffalo [from Ukraine (FJ223614.1) and Mexico (MN811651.1)]; sheep–cattle [from Turkey(ON401193.1) and Czech Republic (EF451586.1), as well as Sweden (U65060.1) and South Korea (MH396616.1)], and alpacas–alpacas [from the USA (FJ387265.1) and Canada (FJ387310.1)] (Figure 2). Based on the N-pro gene phylogenetic analysis buffalo- goat [U80901.1 and U80899.1] in Australia as well as goat-pig (U80900.1-AF144471.1) from New Zealand and Germany showed more than 96% similarity (Figure 3).

Moreover, the BVDV-1 sequence obtained from different wildlife and domestic animal species also found in the same clade indicates cross-species spillover (Figure 2 and Figure 3), such as wild boar–cattle [from Serbia (KY941182.1 and PP657444.1)], yak–cattle [from Mongolia (LC099927.1 and LC099930.1)]; deer–cattle [from Mexico (MN811649.1 and KC252588.1)], and wild boar–cattle [from Brazil and Colombia (MT437382.1 and MH198306.1)]. In China, a high genetic similarity was observed between cattle and buffalo (KJ578865.1- KJ578811.1) (Figure 2). Oceanian countries like Australia and New Zealand reported BVDV-1 infections in goats (U80899.1) and deer (U80903.1), respectively, which were found in a unique phylogenetic clade (Figure 3). Interestingly, in Germany, wild animals such as bison (AF144476.1) and deer (AF144475.1) infected with BVDV-1 have been found within a single clade, showing a high genetic similarity (Figure 3). BVDV-1 isolates from different animal species, including cattle, buffalo, pigs, and deer from India (DQ067601.1), China (FJ555203.1, AF526381.3, KJ578865.1, and KJ578811.1), and Russia (OQ784258.1), clustered together with a high bootstrap value, indicating potential cross-border transmission. Furthermore, cattle isolates from neighboring European countries such as Germany (OR710422.1)–Poland (JN715017.1), Slovakia (AF287278.1)–Austria (AF298065.1), Portugal (EU034183.2)–Spain (AY159534.1), Switzerland (MH901234.1)–Italy (KY040393.1), and Slovenia (AY323890.1)–Croatia (MW057258.1) were found in the same cluster and clade (Figure 2). BVDV-1 isolated from different animal species has also been grouped within the same phylogenetic clade in South and North American countries like Brazil (MT437382.1)–Colombia (MH198306.1) and the USA (FJ387265.1)–Canada (FJ387310.1). Similarly, in Africa, countries like South Africa (U97440.1)–Mozambique (U97423.1), as well as Asian countries such as Iran (EF210347.1)–Iraq (MF347398.1), BVDV-1 isolated from cattle has been found in the same clade with high genetic similarities. The presence of the same BVDV species in neighboring and border-sharing countries provides strong genetic evidence of cross-border and transboundary transmission.

### 3.2. Genetic Variability and Phylogenetic Evidence of BVDV-2 (P. tauri) Intra- and Cross-Species Spillover

BVDV-2 also has a high mutability and plays an identical role in the ecology of BVDV infection among diverse mammalian species in different geographical locations (Figure 1). The phylogenetic analysis showed that all BVDV-2 species were grouped into common clusters and subsequently divided into several sub-clades based on genomic variability originating from different animal species (Figure 2 and Figure 3). BVDV-2 isolates derived from cattle in Poland (JN715017.1), Slovakia (AF287278.1), Israel (BVDV-2 species in cattle), the UK (AF298063.1), and Austria (AF298065.1) were found in a distinct cluster with a high bootstrap value (Figure 2). Moreover, virus sequences in France (AF298055.1)–Japan (AB300662.1), Iraq (MF491394.1)–Switzerland (U94914.1), and Uruguay (MG923951.1)–Argentina (AF244952.1) are found to share a common homology in the phylogenetic group. However, the significant number of cattle-originated BVDV-2 species were aligned with similar clusters and clades of other animal species within the same countries, such as in India for cattle (MF157329.1)–sheep (EU371402.1) and in the USA for goat (FJ431194.1)–pig (AF039174.1), as well as in Italy for cattle (KX350067.1)–goat (KX350069.1)–sheep (KX350071.1), indicating a high likelihood of cross-species transmission. Moreover, the BVDV-2 isolate from cattle in Turkey (MG931953.1) is closely related to an isolate from sheep in Sweden (U65055.1), with both being found in the same clade. BVDV-2 species isolated from cattle in neighboring countries such as Poland (KJ616409.1) and Slovakia (EU747875.1), the USA (AF502399.1) and Canada (AY149215.1), and Uruguay (MG923951.1) and Argentina (AF244952.1), as well as Portugal (AY944277.1) and Spain (sheep: KX369602.1), exhibited high genetic similarity and were found in the same clade.

### 3.3. Genetic Variability and Phylogenetic Evidence of HoBi-like Pestivirus (P. brazilense) Intra- and Cross-Species Spillover

The genomic analysis of various BVDV-3 sequences retrieved from diverse geographic regions (Figure 1) has revealed the presence of a distinct lineage within the BVDV species, distinguished by unique genetic signatures (Figure 2). The phylogenetic tree showed the relatedness of BVDV-3 sequences originating from cattle, such as between India (KM201313.1)–Thailand (DQ897641.1), Turkey (MG948565.1)–Italy (HM151361.1), and Egypt (MZ873106.1)–Argentina (MZ189734.1), as well as between Sweden (JN967724.1) and Australia (JN967728.1). In China, cattle- (MN442384.1), goat- (KU053489.1), and sheep (KU053493.1)-originated BVDV-3 were found in the same clade with a high similarity indicating cross-species infection. Moreover, BVDV-3 infection was identified in distinct animals species in different countries such as sheep (KU053493.1) and goat (KU053489.1) in China, buffalo (PV535837.1, MN537910.1) in Bangladesh and Italy, and sheep (MN537909.1) in Italy, as well as pig (U80905.1) in Netherlands. They were positioned in the same homology of cattle-originated BVDV-3 isolates (Figure 2). Nevertheless, an unusual event such as the BVDV-3 (OR439364.1) sequence, obtained from a bat nasal sample in China, displayed a high level of similarity (>96%) to the BVDV-3 isolates of goat in China (KU563155.1) and cattle in Brazil (KY683847.1), Italy (KJ627179.1), and Japan (AB871953.1) in the Blastn analysis of the polyprotein gene. Another uncertain event is that of the giraffe pestivirus (pestivirus G), which is not limited within the giraffe species and is capable of infecting other animal species, as reported in cattle (OR425033.1) in Egypt, identified in the NCBI GeneBank (Table 1).

### 3.4. Putative BVDV Transmission Cycle

**Figure 4:**
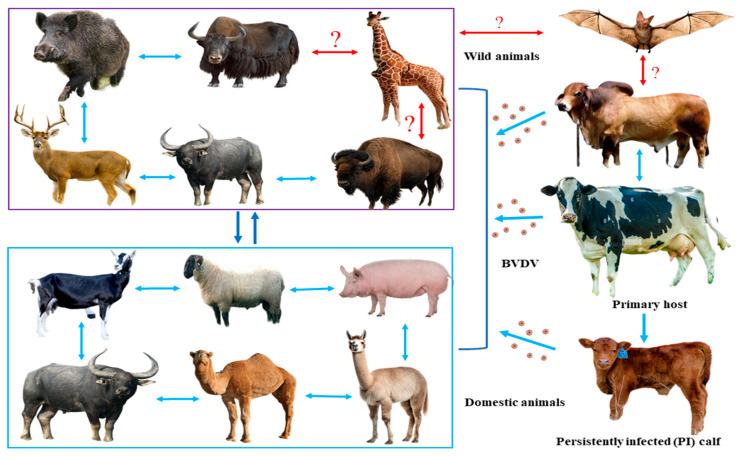
The proposed image depicts the evolution and predicts the cross-species transmission/spillover of BVDV among different domestic and wildlife species. The blue color arrows represent direct (confirmed or suspected) BVDV transmission between intra- and interspecies, as well as among domestic and wild animals. The red arrows with a question mark indicate uncertain spillover events. However, all animal species incorporated in the current transmission dynamics image are taken from published findings as well as NCBI GenBank database information.

Based on the BVDV genome sequences collected from various animal species across diverse habitats (Table 1), we postulated a BVDV transmission pattern (Figure 4) where cattle are the primary host and source of infection, while other domestic and wildlife mammals serve as incidental hosts. Notably, when BVDV-infected domestic animals come into close contact with susceptible wild animals, particularly wild ruminants or pigs, there is the high likelihood of active virus transmission and infection in either direction. However, once infected, all susceptible animal species may contribute to maintaining the infection ecology (Figure 4) due to close contact and the viruses’ host tropism.

## 4. Discussion

A targeted gene (5′-UTR and N-pro) sequence-based phylogenetic analysis was performed using various BVDV species detected from diverse host species across different geographical locations, available in the NCBI GenBank database. The structure of the phylogenetic tree, supported by significant bootstrap values from 1000 replicates, facilitated a dependable interpretation of the genetic relatedness among BVDV infection across various domestic and wildlife species. In current phylogenetic analysis, the branched length and different clusters of cladograms might represent the three BVDV species presumably diverged from their common ancestor, implying a closer genetic relationship with unique genetic signatures and properties. Moreover, the phylogenetic tree depicts that all of the BVDV sequences are divided into different major clusters and clades, namely BVDV-1, BVDV-2, and HoBi-like pestivirus, as classified previously [17,18,19,20]. Over the course of time, this evolutionary genomic diversity is commonly associated with genetic mutations and recombination, as well as error-prone viral RNA replication processes [21,22]. Significant mutations or alterations in particular genes can lead to changes in the BVDV proteins that might affect the virus’s properties in terms of transmissibility, infectivity, pathogenicity, and host range [8].

A unique feature of the phylogenetic tree is that the diversity of BVDV-1, 2, HoBi-like pestivirus is correlated among the different host populations that originated from distinct geographical locations. Moreover, BVDV-1 species isolated from diverse mammalian species are clustered together in the same clade, originating from neighboring border countries such as India-China, Iran–Iraq, Germany–Poland, Portugal–Spain, Switzerland –Italy, Slovakia–Austria, Croatia–Slovenia, Brazil–Colombia, the USA–Canada, and South Africa–Mozambique, as well as New Zealand–Australia. Similarly, BVDV-2 isolated from Poland–Slovakia, Portugal–Spain, Uruguay–Argentina, and the USA–Canada and BVDV-3 from Mexico–USA and Bangladesh–India–China aligned along side-by-side borders countries. This scenario strongly indicates that cross-border transmission of BVDV may occur through the exchange of BVDV-positive animals during export and import [23], sharing of common pasture lands, movement of free-range reservoir animals across borders, and importation of animal-derived biological products like milk, semen, bovine fetal serum, meat, hide and skin, etc. [24]. Moreover, the current sequence-based analysis provides strong evidence that BVDV has a high host plasticity and can infect a wide range of domestic as well as wildlife mammals. In addition to cattle, other bovine species like buffalo (*Bubalus bubalis*), bison (*Bison bison*), and yak (*Bos grunniens*) were also found to be infected with different species of BVDV [1,6]. Surprisingly, the small ruminants like sheep, goats, and deer, as well as the pseudo-ruminants (members of the camelid family), like camel and alpaca, are infected with different strains of BVDV [6,7]. In addition, monogastric animals like pig and wild boar carried BVDV in different countries, positioned in the same linage of BVDV that originated from cattle isolates in the phylogenetic tree with a high degree of genomic similarity. Notably, the cross-species spillover phenomenon of pestiviruses, especially BVDV, has been previously reported in different studies [1,6,9]. Another notable finding from the present analysis is that the BVDV sequence is derived from the same country, but their sources of isolation, like the infected host species, are different. For instance, in the current phylogenetic analysis, both wild boar and cattle in Serbia and domestic sheep and goat, as well as cattle and buffalo in China, sheep and buffalo in Argentina, deer and cattle in Mexico, yak and cattle in Mongolia, alpacas and cattle in Canada, and goat and buffalo in Australia infected with BVDV-1 were found in the same clade of the cladogram. Similarly, in India, cattle and sheep; in the USA, goat and pig; and in Italy, cattle, sheep, and goat were infected with BVDV-2, and their sequences were placed in the same clade with high genetic similarity in the phylogenetic tree. Moreover, cattle, goat, and sheep in China, as well as cattle and buffalo in Italy, infected with BVDV-3 were found in a similar lineage with a high degree of genetic relatedness. This genetic evidence indicates the possibility of host jumping or cross-species spillover of BVDV among various host species across different countries or within the same countries. Such cross-species transmission might occur in mixed farming conditions, where different animal species are reared together with persistently infected animals, sharing common pasture lands, and using shared management utensils, which are closely similar biosecurity practices as reported in previous studies [25,26]. Although cattle are the primary host, detection of BVDV in other domestic and wildlife hosts is uncommon and often incidental, typically reported during investigations of reproductive or respiratory disorders or through routine surveillance in many countries [1,2,6]. Such findings may introduce a species bias; therefore, we recommend long-term, broad-scale molecular surveillance of BVDV in both domestic and wildlife populations to generate epidemiological and virological evidence. Additionally, the detection of BVDV-specific antibodies in diverse domestic and wildlife species indicates previous exposure and potential cross infection [6,17]. However, isolation or molecular detection of the active virus is not always possible, primarily due to challenges in sampling animals during the viremic phase or when sampling takes place at later stages of infection.

Besides domestic animals, several captive and free-range wild species, namely wild boar, deer, wild buffalo, yak, and bison, have been documented with a BVDV infection, and their genomic sequences bear a close resemblance to cattle isolates in the current study. The close contact between domestic and wild animals may facilitate viral spillover and spillback events, thereby broadening the host range and complicating control efforts. Similar findings have been reported previously by several researchers [1,6,9,27,28]. BVDV has a broader cell tropism, and it can infect and replicate in a wide range of cell types across different animal species [29,30]. The majority of large and small ruminants possess closely similar receptor-binding domains for BVDV, which may facilitate viral host tropism. BVDV primarily exploits the complement regulatory protein 46 (CD46) and glycosaminoglycans as cellular receptors to facilitate viral entry, [31] and the presence of this receptor on cells of different species allows BVDV to infect a wide range of hosts beyond cattle. Moreover, BVDV can also interact with different host cell receptors like heparan sulfate (HS), the low-density lipoprotein (LDL) receptor, and disintegrin and metalloproteinase 17 (ADAM17) receptors for primary attachment and entry into host cells [29,32,33]. Among host cell factors, ADAM17 is the only essential molecule reported to mediate pestivirus entry, including both CSFV and BVDV [31,33]. Unfortunately, this ability facilitated BVDV to utilize different receptors that are present on the cells of diverse animal species and could be responsible for the high host plasticity. In addition, PI is an important feature of BVDV, where the virus can persistently replicate within the host cells and continue to shed abundant copies of active viruses [34]. This persistence may also facilitate the transmission of the virus to other susceptible animals, including different animal species. It is important to note that the infection status of canine, feline, and equine species with BVDV in wildlife environments remains largely unreported. Therefore, the potential impact of BVDV on these animals and the role they may play in the virus’s transmission cycle are not well understood. Thus, it is difficult to understand the definite reservoir or carrier of BVDV in wildlife conditions maintaining the infection ecology. These transmission patterns highlight the need for integrated surveillance and biosecurity measures at the livestock–wildlife interface to prevent cross-species spread and sustain long-term control strategies. Further research is highly recommended to assess the prevalence and effects of BVDV in these species within wildlife settings.

Although several countries effectively vaccinate cattle herds against BVDV [24,34], many regions still do not use vaccination as a preventive measure [1,3,26]. Lacking in vaccinating other animal species, including wildlife, allows BVDV strains to circulate freely, increasing the likelihood of spillover into new hosts and viral adaptation. Historically, a few countries occasionally used BVDV live vaccines to protect swine against the classical swine fever virus (CSFV) [35]. Studies demonstrate that BVDV antibodies can reduce CSFV excretion and may also limit or prevent the transmission of highly virulent CSFV strains [36], likely due to the antigenic relatedness between BVDV and CSFV, which may provide cross-protection. However, using such cross-protective live vaccines in different hosts may increase host susceptibility and viral adaptability, creating opportunities for viral reassortment and the emergence of novel strains [21,37]. Notably, various biological products such as bovine fetal serum, cell cultures, vaccines, and bull semen used for breeding purposes have been found to be contaminated with different pestivirus species, including BVDV-1, BVDV-2, and HoBi-like pestivirus [38]. The introduction of such contaminated products into the host body can result in unnoticed infections and poses a significant risk for global spread and distribution. We carefully excluded sequences that did not represent true animal infections. Nonetheless, these sequences remain epidemiologically significant, as they may reflect cross-border or transboundary transmission events of BVDV.

In this review, we identified certain unusual events, such as the detection of HoBi-like pestivirus from bat species in China and its genetic similarity to ruminant BVDV strains, and the infection of cattle with a giraffe pestivirus (*P. giraffae*) that is naturally harbored by giraffes [4]. This unusual case might be a concern for future noteworthy transmission patterns as well as evolutionary dynamics. Although the giraffe pestivirus was initially regarded as a HoBi-like pestivirus [17], recent genome-based classification has identified it as a novel species, namely *P. giraffae* (previously pestivirus G) [17,39]. It is alarming that, if BVDV continues to adapt within mammalian species, there is the potential risk of the establishment of infection in new reservoirs or amplification in carrier hosts, given that the virus receptors are also present on almost all mammalian host cell surfaces [31,32,33]. Although no evidence has been found to date, an unexpectedly significant mutation of the BVDV nucleotide may be anticipated to alter potential host–pathogen interactions and could be suspected of facilitating animal-to-human transmission, possibly leading to emerging zoonosis. Hence, a contentious molecular survey among domestic and wild host species with a high-depth, refined core genomic study is highly recommended to understand the molecular epidemiology and evolutionary phylodynamics of BVDV. The virus is constantly evolving and adapting; hence, the emergence of new variants may occur especially in mixed-species farming environments where co-infection with different host–origin virus strains is plausible [37]. Investing in research to develop genotype/subgenotype-specific vaccines that confer lifelong immunity is essential for BVDV control. Exploring heritable traits in livestock that reduce susceptibility, limit viral replication, and confer tolerance to BVDV can enhance herd resilience. Moreover, with the application of CRISPR-mediated editing and somatic cell nuclear transfer, a calf with a six-amino acid substitution in the BVDV-binding domain of CD46 has been produced, showing a markedly reduced susceptibility evidenced by the absence of clinical signs and viral infection in white blood cells [40]. However, to date, persistent infection (PI) has been well-documented in cattle [2], the primary host of BVDV; however, PI has also been reported in goats, pigs, and deer [41,42,43]. The occurrence of PI across multiple host species complicates transmission dynamics and poses significant challenges for the complete eradication of BVDV. Therefore, implementing early screening and culling of PI animals from animal herds provides a sustainable, long-term strategy for controlling the virus. However, although the 5′ UTR and N-pro genes provide useful insights, their short length limits the resolution for determining host range. The detection of identical viral sequences across different species, along with the clustering of 5′ UTR and N-pro sequences from diverse hosts, is evidence of their genetic resemblance and supports the hypothesis of cross-species transmission by the same virus. Therefore, broader genomic studies are needed to elucidate how genetic changes enable BVDV to adapt to new hosts.

## 5. Conclusions

The current review represents and summarizes the host-specific, viral sequence-based genetic diversity and distribution of BVDV across the world, highlighting the variability of BVDV interactions and potential cross-species spillover among diverse host species. The phylogenetic analysis strongly indicates that all BVDV species have the potential ability to infect a wide range of domestic animals such as cattle, buffalo, sheep, goat, pig, camel, and alpacas, as well as wild mammalian hosts such as deer, yak, bison, wild boar, giraffe, and bats. The virus shows high host plasticity due to high tissue tropism and divergent cell receptor affinity across animal species and potential cross-species transmission among the mammalian species. Therefore, interconnected roles of different animal species within shared environments along with the environmental stability of the virus are necessary to explore. Standardizing biosecurity protocols and fostering cross-species collaboration are vital for a unified approach to disease management. Nevertheless, this is the first sequence-based review study highlighting host plasticity and exploring the potential of cross-species spillover of BVDV in domestic and wildlife environments.

## 6. Limitations of the Study

The current review has considered a single sequence from individual countries from each BVDV-positive animal species. This limitation is inevitable due to the sequence selection strategy, which included only a single sequence per BVDV species per host for every country to reduce alignment bias and minimize redundancy. Additionally, partial sequences are analyzed. It is worth mentioning that, as the majority of countries have not performed whole-genome sequencing for BVDV; instead, the majority of submissions to GenBank consist of partial sequences. This approach may limit the representation of the broad genetic diversity of BVDV within specific countries and animal species. Therefore, detailed bioinformatic analysis using a complete genome to identify the specific viral protein that interacts with different host cell receptors, enhancing the spillover events among diverse animal species, is necessary. Additionally, the exclusion of molecularly confirmed but non-sequenced cases may overlook important evidence of cross-species transmission, particularly in countries with limited laboratory and sequencing facilities. These limitations should be addressed, with a focus on detailed genomic insights of different BVDV species in future review studies.

## Figures and Tables

**Table 1:** List of 5’-UTR and N-pro fragmented sequences of different BVDV species isolated from different domestic and wild animal species, retrieved from NCBI GenBank database.

Continent	Country	BVDV Species	Targeted Genes	Host	GenBank Accession Number
	Bangladesh	BVDV-1	5′-UTR	Cattle	PV345782.1
		BVDV-2	5′-UTR	Cattle	PV580259.1
		BVDV-3	5′-UTR	Cattle	KF204448.1
		BVDV-1	5′-UTR	Buffalo	PV492126.1
		BVDV-2	5′-UTR	Buffalo	PV504831.1
		BVDV-3	5′-UTR	Buffalo	PV535837.1
	China	BVDV-1	5′-UTR	Cattle	KJ578865.1
		BVDV-1	5′-UTR	Pig	AF526381.3
		BVDV-1	5′-UTR	Goat	KP749801.1
		BVDV-1	5′-UTR	Buffalo	KJ578811.1
		BVDV-1	5′-UTR	Yak	KJ578902.1
		BVDV-1	5′-UTR	Camel	JX276538.1
		BVDV-1	5′-UTR	Deer	FJ555203.1
		BVDV-2	5′-UTR	Cattle	OP792028.1
		BVDV-3	5′-UTR	Cattle	MN442384.1
		BVDV-3	5′-UTR	Sheep	KU053493.1
		BVDV-3	5′-UTR	Goat	KU053489.1
		BVDV-3	Poly protein gene	Bat	OR439364.1
	India	BVDV-1	5′-UTR	Cattle	KM201317.1
		BVDV-1	5′-UTR	Pig	KY886197.1
		BVDV-1	5′-UTR	Goat	JQ679447.1
		BVDV-1	5′-UTR	Buffalo	DQ067601.1
		BVDV-2	5′-UTR	Cattle	MF157329.1
		BVDV-2	5′-UTR	Sheep	EU371402.1
		BVDV-3	5′-UTR	Cattle	KM201313.1
	Indonesia	BVDV-1	5′-UTR	Cattle	MK411762.1
	Iran	BVDV-1	5′-UTR	Cattle	EF210347.1
	Iraq	BVDV-1	5′-UTR	Cattle	MF347398.1
		BVDV-2	5′-UTR	Cattle	MF491394.1
	Israel	BVDV-2	5′-UTR	Cattle	BVDV-2 (Not submitted in GeneBank)
	Japan	BVDV-1	5′-UTR	Cattle	OM238259.1
		BVDV-2	5′-UTR	Cattle	AB300662.1
		BVDV-3	5′-UTR	Cattle	AB871953.1
	Kazakhstan	BVDV-1	5′-UTR	Cattle	OP650999.1
		BVDV-2	5′-UTR	Cattle	OQ451771.1
	Kosovo	BVDV-1	5′-UTR	Cattle	BVDV-1135 (Not submitted in GeneBank)
	Mongolia	BVDV-1	5′-UTR	Cattle	LC099930.1
		BVDV-1	5′-UTR	Yak	LC099927.1
		BVDV-2	5′-UTR	Cattle	LC099932.1
		BVDV-2	5′-UTR	Yak	LC099925.1
	Malaysia	BVDV-2	5′-UTR	Pig	MH814636.1
	South Korea	BVDV-1	5′-UTR	Cattle	MH396616.1
		BVDV-2	5′-UTR	Cattle	MH396619.1
		BVDV-2	5′-UTR	Goat	DQ973184.1
	Thailand	BVDV-3	5′-UTR	Cattle	DQ897641.1
Europe	Austria	BVDV-1	5′-UTR	Cattle	AF298065.1
		BVDV-2	5′-UTR	Cattle	EU224225.1
	Belgium	BVDV-1	5′-UTR	Cattle	KU200260.1
	Croatia	BVDV-1	5′-UTR	Cattle	MW057258.1
	Czech Republic	BVDV-1	5′-UTR	Cattle	EF451586.1
	Denmark	BVDV-1	5′-UTR	Cattle	AY363072.1
		BVDV-1	5′-UTR	Deer	AY158154.1
	France	BVDV-1	5′-UTR	Cattle	ON155755.1
		BVDV-2	5′-UTR	Cattle	AF298055.1
	Germany	BVDV-1	5′-UTR	Cattle	OR710422.1
		BVDV-1	N^pro^	Goat	U80898.1
		BVDV-1	N^pro^	Bison	AF144476.1
		BVDV-1	N^pro^	Deer	AF144475.1
		BVDV-1	N^pro^	Pig	AF144471.1
		BVDV-2	5′-UTR	Cattle	AY379547.1
		BVDV-3	N^pro^	Cattle	AY735486.1
	Italy	BVDV-1	5′-UTR	Cattle	KY040393.1
		BVDV-2	5′-UTR	Cattle	KX350067.1
		BVDV-2	5′-UTR	Sheep	KX350071.1
		BVDV-2	5′-UTR	Goat	KX350069.1
		BVDV-3	5′-UTR	Cattle	HM151361.1
		BVDV-3	5′-UTR	Buffalo	MN537910.1
		BVDV-3	5′-UTR	Sheep	MN537909.1
	Ireland	BVDV-1	5′-UTR	Cattle	AJ312927.1
	Netherlands	BVDV-3	5′-UTR	Pig	U80905.1
	Poland	BVDV-1	5′-UTR	Cattle	JN715017.1
		BVDV-2	5′-UTR	Cattle	KJ616409.1
	Portugal	BVDV-1	5′-UTR	Cattle	EU034183.2
		BVDV-2	5′-UTR	Cattle	AY944277.1
	Russia	BVDV-1	5′-UTR	Cattle	OQ784258.1
	Serbia	BVDV-1	5′-UTR	Cattle	PP657444.1
		BVDV-1	5′-UTR	Wild Boar	KY941182.1
	Slovakia	BVDV-1	5′-UTR	Cattle	AF287278.1
		BVDV-2	5′-UTR	Cattle	EU747875.1
	Slovenia	BVDV-1	5′-UTR	Cattle	AY323890.1
	Spain	BVDV-1	5′-UTR	Cattle	AY159534.1
		BVDV-2	5′-UTR	Sheep	KX369602.1
	Switzerland	BVDV-1	5′-UTR	Cattle	MH901234.1
		BVDV-2	5′-UTR	Cattle	U94914.1
	Sweden	BVDV-1	5′-UTR	Sheep	U65060.1
		BVDV-1	5′-UTR	Cattle	U65029.1
		BVDV-2	5′-UTR	Sheep	U65055.1
		BVDV-3	5′-UTR	Cattle	JN967724.1
	Turkey	BVDV-1	5′-UTR	Cattle	MG973218.1
		BVDV-1	5′-UTR	Sheep	ON401193.1
		BVDV-2	5′-UTR	Cattle	MG931953.1
		BVDV-3	5′-UTR	Cattle	MG948565.1
	Ukraine	BVDV-1	5′-UTR	Cattle	FJ223614.1
	United Kingdom (UK)	BVDV-1	5′-UTR	Cattle	LT902259.1
		BVDV-1	5′-UTR	Sheep	U65053.1
		BVDV-1	N^pro^	Deer	U80902.1
		BVDV-2	5′-UTR	Cattle	AF298063.1
North America	Canada	BVDV-1	5′-UTR	Cattle	KX170315.1
		BVDV-1	5′-UTR	Alpacas	FJ387310.1
		BVDV-2	5′-UTR	Cattle	AY149215.1
		BVDV-3	5′-UTR	Cattle	JN967711.1
	Costa Rica	BVDV-1	5′-UTR	Cattle	MT024564.1
	Mexico	BVDV-1	5′-UTR	Cattle	KC252588.1
		BVDV-1	5′-UTR	Buffalo	MN811651.1
		BVDV-1	5′-UTR	Deer	MN811649.1
		BVDV-2	5′-UTR	Cattle	JN967713.1
		BVDV-3	5′-UTR	Cattle	JN967747.1
	United States of America (USA)	BVDV-1	5′-UTR	Cattle	FJ387311.1
		BVDV-1	5′-UTR	Goat	FJ431195.1
		BVDV-1	5′-UTR	Pig	MT265675.1
		BVDV-1	5′-UTR	Alpacas	FJ387265.1
		BVDV-2	5′-UTR	Cattle	AF502399.1
		BVDV-2	5′-UTR	Goat	FJ431194.1
		BVDV-2	5′-UTR	Pig	AF039174.1
		BVDV-3	5′-UTR	Cattle	JN967748.1
South America	Argentina	BVDV-1	5′-UTR	Cattle	AF418000.1
		BVDV-1	5′-UTR	Buffalo	FM165316.1
		BVDV-1	5′-UTR	Sheep	FJ615532.1
		BVDV-2	5′-UTR	Cattle	AF244952.1
		BVDV-2	5′-UTR	Buffalo	FM165309.1
		BVDV-3	5′-UTR	Cattle	MZ189734.1
	Brazil	BVDV-1	5′-UTR	Cattle	KF023365.1
		BVDV-1	5′-UTR	Wild Boar	MT437382.1
		BVDV-3	5′-UTR	Cattle	MK495429.1
		BVDV-2	5′-UTR	Cattle	JN967743.1
	Chile	BVDV-1	5′-UTR	Cattle	OL860954.1
		BVDV-2	5′-UTR	Cattle	AF356505.1
	Colombia	BVDV-1	5′-UTR	Cattle	MH198306.1
	Uruguay	BVDV-1	5′-UTR	Cattle	OR620203.1
		BVDV-2	5′-UTR	Cattle	MG923951.1
	Panama	BVDV-2	N^pro^	Cattle	AF144468.1
Africa	Algeria	BVDV-1	5′-UTR	Cattle	MT157227.1
	Ethiopia	BVDV-2	5′-UTR	Cattle	PQ096502.1
	Egypt	BVDV-1	5′-UTR	Cattle	KP127973.1
		BVDV-3	5′-UTR	Cattle	MZ873106.1
		Pestivirus Giraffe-2	5′-UTR	Cattle	OR425033.1
	Mozambique	BVDV-1	5′-UTR	Cattle	U97423.1
	South Africa	BVDV-1	5′-UTR	Cattle	U97440.1
	Tanzania	BVDV-1	N^pro^	Cattle	OU548655.1
	Tunisia	BVDV-1	5′-UTR	Cattle	AY453631.1
		BVDV-2	5′-UTR	Cattle	AF462005.1
Oceania	Australia	BVDV-1	5′-UTR	Cattle	AY763092.1
		BVDV-1	N^pro^	Buffalo	U80901.1
		BVDV-1	N^pro^	Goat	U80899.1
		BVDV-3	5′-UTR	Cattle	JN967728.1
	New Zealand	BVDV-1	5′-UTR	Cattle	OR239051.1
		BVDV-1	N^pro^	Deer	U80903.1
		BVDV-1	N^pro^	Goat	U80900.1

## Data Availability

The sequence datasets used and analyzed in the current review study are available in the manuscript.

## Abbreviations

The following abbreviations are used in this manuscript:

BVDV	Bovine Viral Diarrhea Virus
BVDV-1	Bovine Viral Diarrhea Virus-1
BVDV-2	Bovine Viral Diarrhea Virus-2
CSFV	Classical Swine Fever Virus
UTR	Untranslated Region
N-pro	N-terminal Autoprotease
NCBI	National Center for Biotechnology Information
